# A Combined Mass Spectrometry and Data Integration Approach to Predict the Mitochondrial Poly(A) RNA Interacting Proteome

**DOI:** 10.3389/fcell.2019.00283

**Published:** 2019-11-15

**Authors:** Selma L. van Esveld, Şirin Cansız-Arda, Fenna Hensen, Robin van der Lee, Martijn A. Huynen, Johannes N. Spelbrink

**Affiliations:** ^1^Radboud Center for Mitochondrial Medicine, Center for Molecular and Biomolecular Informatics, Radboud Institute for Molecular Life Sciences, Radboud University Medical Centre, Nijmegen, Netherlands; ^2^Department of Pediatrics, Radboud Center for Mitochondrial Medicine, Radboud University Medical Centre, Nijmegen, Netherlands

**Keywords:** mitochondrial RNA, 4-thiouridine labeling and crosslinking, Bayesian data integration, mass spectrometry, proteome

## Abstract

In order to synthesize the 13 oxidative phosphorylation proteins encoded by mammalian mtDNA, a large assortment of nuclear encoded proteins is required. These include mitoribosomal proteins and various RNA processing, modification and degradation enzymes. RNA crosslinking has been successfully applied to identify whole-cell poly(A) RNA-binding proteomes, but this method has not been adapted to identify mitochondrial poly(A) RNA-binding proteomes. Here we developed and compared two related methods that specifically enrich for mitochondrial poly(A) RNA-binding proteins and analyzed bound proteins using mass spectrometry. To obtain a catalog of the mitochondrial poly(A) RNA interacting proteome, we used Bayesian data integration to combine these two mitochondrial-enriched datasets as well as published whole-cell datasets of RNA-binding proteins with various online resources, such as mitochondrial localization from MitoCarta 2.0 and co-expression analyses. Our integrated analyses ranked the complete human proteome for the likelihood of mtRNA interaction. We show that at a specific, inclusive cut-off of the corrected false discovery rate (cFDR) of 69%, we improve the number of predicted proteins from 185 to 211 with our mass spectrometry data as input for the prediction instead of the published whole-cell datasets. The chosen cut-off determines the cFDR: the less proteins included, the lower the cFDR will be. For the top 100 proteins, inclusion of our data instead of the published whole-cell datasets improve the cFDR from 54% to 31%. We show that the mass spectrometry method most specific for mitochondrial RNA-binding proteins involves *ex vivo* 4-thiouridine labeling followed by mitochondrial isolation with subsequent *in organello* UV-crosslinking.

## Introduction

Human mitochondrial DNA (mtDNA) has limited coding capacity, having only 37 genes coding for 13 subunits of oxidative phosphorylation enzyme complexes, 22 transfer RNAs (tRNAs) and 2 ribosomal RNAs (rRNAs). In order to synthesize the 13 mtDNA encoded OXPHOS subunits, a unique mtDNA replication, transcription and translation system is in place. This system requires the combined action of mtDNA, its structural RNA components and as many as 250–300 nuclear-encoded gene products (Pearce et al., 2017) that are translated on cytosolic ribosomes and imported into mitochondria by dedicated outer- and inner-membrane machineries. The majority of these imported proteins are directly or indirectly involved in mitochondrial translation. The mitoribosome alone already contains some 80 proteins, while its assembly requires additional factors such as rRNA modifying enzymes. Likewise, mitochondrial tRNAs, similar to cytosolic tRNAs, are heavily modified and in addition need post-transcriptional processing and maturation, as do mitochondrial mRNAs and rRNAs, as a consequence of their initial synthesis as polycistronic transcripts. Therefore, a functional mitochondrial gene expression system requires the action of a large variety of mitochondrial RNA interacting proteins.

So far, over 80 proteins of the mitochondrial gene expression system have been implicated in mitochondrial diseases, which led us to use comparative proteomic approaches following various targeted purification strategies in order to identify the proteins involved. In the past, we applied whole-cell crosslinking combined with Twinkle helicase directed purification of mitochondrial nucleoids (Rajala et al., 2015). This method identified a large set of proteins associated not only with mtDNA maintenance but also gene expression, and did not allow us to distinguish *a priori* between for example mtDNA maintenance proteins and RNA associated proteins. Whole-cell RNA crosslinking in recent years has identified large sets of cellular RNA binding proteins (Baltz et al., 2012; Castello et al., 2012), including a substantial set of mitochondrial RNA binding proteins (Zaganelli et al., 2017). However, these approaches were not specifically targeted to mitochondria.

Here we describe and compare two mass spectrometry based approaches applied specifically to identify the mitochondrial poly(A) RNA binding proteome: (i) using either whole-cell crosslinking followed by mitochondrial and poly(A) mtRNA isolation, or (ii) using crosslinking after mitochondrial isolation (mitochondrial crosslinking) and followed by poly(A) mtRNA isolation. Application of Bayesian statistics comparing our own mass spectrometry data with published mass spectrometry data sets made it apparent that mitochondrial crosslinking is the most efficient method to specifically enrich mitochondrial proteins known to interact with mtRNA and leads to the lowest level of cytosolic protein contamination. In terms of both relative and absolute number of identified mitochondrial proteins, mitochondrial crosslinking outperformed whole-cell crosslinking followed by mitochondrial isolation. Nevertheless, the latter method still enriched more for mitochondrial proteins when compared to published whole-cell RNA-binding proteomes (Baltz et al., 2012; Castello et al., 2012). We have used both methods to identify mitochondrial poly(A)-RNA binding proteomes and have combined them with various publicly available datasets, such as MitoCarta 2.0 and co-expression data, using Bayesian data integration to obtain a statistically founded list of poly(A) mtRNA interacting proteins.

## Materials and Methods

### Reference Human Proteome

Throughout all analyses, the human proteome from the reviewed UniProtKB/Swiss-Prot database release 2016_11 (The UniProt Consortium, 2018) was used as the reference proteome. This version consists of 20129 entries, in which each entry refers to all protein products encoded by a single gene, so including isoforms the database contains 42111 proteins. All used datasets were mapped to the reference proteome, using the mapping table from the same UniProt release, ambiguities were checked manually.

### Cell Culture

HEK293e cells (ATCC CRL-1573) were grown in Dulbecco’s modified Eagle’s medium (DMEM; Lonza BE12- 604F) supplemented with 10% fetal calf serum (GE Healthcare) in a 37°C incubator at 5% CO_2_. Cells were frequently tested for mycoplasma contamination and found to be negative. When required, cells were incubated for indicated time periods with indicated concentrations of ethidium bromide to deplete the cells of mitochondrial RNA and/or for 18 h with 100 μM 4-thiouridine to enhance crosslinking efficiency. For whole-cell crosslinking conditions, medium was removed from the monolayer of living cells and cells were exposed to 302 nm UV light for 1 min in a ChemiDoc instrument (Bio-Rad).

### Mitochondrial Extraction

Cell pellets were resuspended in hypotonic buffer (4 mM Tris–HCl pH 7.8, 2.5 mM NaCl, 0.5 mM MgCl2, and 2.5 mM PMSF) and incubated for 6 min on ice. The swollen cells were disrupted by 20 strokes with a Dounce homogenizer. Isotonic levels were restored by the addition of 1/10 v/v hypertonic buffer (400 mM Tris–HCl pH 7.8, 250 mM NaCl, and 50 mM MgCl2), and pellets of nuclei and cell debris were discarded by low speed centrifugation (1,200 × *g*). Mitochondria were obtained by centrifugation of the remaining supernatant at 13,000 × *g*. Mitochondria for mitochondrial crosslinking conditions were further purified using a sucrose gradient of 1.0 M and 1.5 M. After centrifugation for 20 min at 60,000 × *g*, the mitochondrial layer was collected, resuspended in PBS, exposed to 302 nm UV light for 6 min in a ChemiDoc instrument (Biorad) and collected by centrifugation at 13,000 × *g*. Protein content of mitochondrial pellets was measured using the Quick Start^TM^ Bradford Protein Assay Kit 2 (Biorad, 5000202) according to manufacturer’s protocol.

### Isolation of the mtRNA Interacting Proteome

The magnetic mRNA Isolation kit (New England Biolabs, S1550S) company protocol was followed in order to extract poly(A) RNA, with the exception that all steps were performed on ice and the incubation of samples with the beads was extended for whole-cell crosslinking to 45 min. In case of whole-cell crosslinking, purification was performed in three rounds to allow all RNA species to bind. In short, mitochondrial pellets were lysed and 2.5–5.5 mg of protein per condition was added to oligo(dT) beads. Stringent washes under denaturing conditions were performed to get rid of non-crosslinked interacting molecules. After elution of the poly(A) RNA with crosslinked proteins attached, RNA was removed by RNase treatment [mitochondrial UV-crosslinking (MXL): RNase A (Thermo Fisher Scientific, EN0531), whole-cell UV-crosslinking (WCXL): RNase A & T1 (Thermo Fisher Scientific, AM2286)]. Remaining mitochondrial RNA interacting proteins were concentrated with Amicon 3K filters (Merck Millipore, UFC500396) and analyzed using SDS-PAGE combined with Western blot and/or mass spectrometry.

### SDS-PAGE and Western Blot Analysis

Proteins were separated by SDS-PAGE and transferred to a nitrocellulose membrane. The blots were probed with primary antibodies to known mitochondrial RNA-interacting proteins; LRPPRC (Abcam, ab21864, 1:1000), POLRMT (Abcam, ab32988, 1:5000), SUV3 (kind gift of Dr. Roman Szczesny, 1:5000) and GRSF1 (Sigma, HPA036985, 1:5000), bound by HRP-conjugated secondary antibodies (Vector Laboratories, 1:10000) and detected by ECL in a ChemiDoc instrument (Biorad).

### Mass Spectrometry Analysis

Proteins were processed as described in the Supplementary Materials and Methods and analyzed with LC-MS/MS in a Q-Exactive mass spectrometer (Thermo Fisher Scientific). Spectra were matched against the reference proteome including isoforms using MaxQuant (version 1.5.0.25) (Cox and Mann, 2008). Further details can be found in the Supplementary Materials and Methods.

Label-free-quantification (LFQ) intensities from the MaxQuant ProteinGroups.txt output file were log2-transformed. Infinite intensities (from missing values) were replaced with the lowest log2(LFQ) measured and the mean of technical and biological repeats was calculated. The difference in log2(LFQ) intensity [=log2(fold-change)] between crosslinking and control conditions (non-crosslinking for MXL and WCXL and crosslinking with EtBr for WCXL_EtBr) was calculated separately for each approach. This difference was used to compare and combine our results with the reported fold-changes of Castello et al. (2012) (we calculated the mean log2(FC) from the three reported experiments 2× XL, 1 × 4SU + XL) and Baltz et al. (2012) [we used the reported mean log2(FC)].

### Data Integration

We integrated five datasets that describe different properties of mitochondrial RNA interacting proteins to distinguish genes encoding these proteins from other genes using a Bayesian statistics approach. We have used this approach successfully to identify genes involved in anti-viral immune responses (van der Lee et al., 2015) and to identify proteins from the gametocyte life stage in the malaria parasite (Meerstein-Kessel et al., 2018). We chose Bayesian integration as it is the most transparent form of data integration that still exploits the relative predictive values of the various datasets and in which the individual contribution of each dataset to a prediction can be observed. Table 1 provides a summary of the datasets used in this study for the final proteome presented, including MitoCarta 2.0 (Calvo et al., 2016) that also used Bayesian data integration for mitochondrial proteome prediction.

**TABLE 1:** Input datasets for Bayesian integration.

**Dataset**	**Description**	**References**	**Number of genes^a^**	**Likelihood mtRNA score^a,b^**
mtRNA interaction	Average fold-change crosslinking with 4SU over non-crosslinking from mitochondrial and whole cell crosslinking experiments	This paper	275	4.16
Mitochondrial localization	Human MitoCarta 2.0 likelihood scores of mitochondrial localization	Calvo et al., 2016	2920	0.81
RNA binding domain	The published list of Pfam domains that are known to be RNA-binding or exclusively found in RNA-related proteins is mapped to UniProt identifiers to determine which human proteins contain an RNA binding domain.	Gerstberger et al., 2014; Finn et al., 2016	1520	1.33
Co-expression with mtRNA interactors	Weighted co-expression with known mtRNA interacting proteins^c^ calculated using WeGET.	Szklarczyk et al., 2016	1500	0.82
PPI with mtRNA interactors	Bioplex database of protein-protein interactions is used to determine which proteins interact with known mtRNA interacting proteins^c^. Per protein the number of mtRNA interacting proteins was counted.	Huttlin et al., 2017	1400	2.01

*^*a*^Based on a combination of genes in all bins with a positive likelihood score. ^*b*^Known mtRNA interacting proteins versus non-mtRNA interacting proteins, see section “Materials and Methods” for calculation. The larger the score, the higher the predictive value of the dataset. ^*c*^Mitochondrial RNA interacting protein list, 218 proteins, contains proteins with both an RNA binding (GO:0003723)and a mitochondrial localization (GO:0005739) GO-term, excluding 24 positive set members and AKAP1 and AUH to avoid circularity. Protein lists were downloaded using the online AmiGO tool (Ashburner et al., 2000; Carbon et al., 2009; The Gene Ontology Consortium, 2017).*

To assess the predictive value of each dataset, a positive and a negative training set was constructed. The positive set contained 24 genes that were included when interaction with mitochondrial mRNA, tRNA or rRNA was described in literature (Supplementary Table S1), structural components of the mitochondrial ribosome were excluded as our focus was on mitochondrial RNA metabolism and not on translation.

As publications providing evidence that a protein is not mitochondrial and/or does not interact with RNA were scarce, we used AND/OR logical combinations of genes associated with four GO-terms (Ashburner et al., 2000; The Gene Ontology Consortium, 2017); molecular function RNA binding GO-term GO:0003723, cellular component mitochondrion GO-term GO:0005739, biological process GO-terms for tricarboxylic acid cycle (TCA) GO:0006099 and oxidative phosphorylation (OXPHOS) GO:0006119. Associated genes were obtained with the online AmiGO tool (Carbon et al., 2009) and were used to create two categories of genes that were combined to form the negative set of 248 genes. The first category contains 124 genes that are mitochondrial and unlikely to interact with mitochondrial RNA. These genes are linked to the mitochondrial GO-term and either the TCA or the OXPHOS GO-term, but not to the RNA-binding GO-term. The TCA and OXPHOS GO-terms were chosen as these are well-studied proteins groups that are unlikely to additionally interact with mtRNA. The second category consists of genes with the RNA binding GO-term, but without the mitochondrial GO-term. In total 1617 genes fall in this category, a large number compared to the 124 in the other category. As we wanted to have an equal contribution of both negative sets to our prediction we randomly selected 124 non-mitochondrial RNA interaction proteins, which together with the 124 TCA/OXPHOS genes, form the negative training set.

For each human protein coding gene a score for mitochondrial RNA interaction was calculated, the higher the score, the more likely it is that the protein product interacts with mitochondrial RNA. In order to calculate these scores, each dataset is separated into several bins and for each bin the presence of training set genes is counted. The log ratio of these counts determines the score for all other genes in the respective bin. The integrated mtRNA score is based on a sum of log ratios of the individual datasets and is calculated as follows:

$$\mathrm{mtRNAscore}=\log_{2}\,\left(\frac{P_{\text{mtRNA}}}{P_{\mathrm{non}–\mathrm{mtRNA}}}\right)+\sum_{i=1}^{n}{\text{log}}_{2}\,\left(\frac{P\,\left({\text{data}}_{i}|\mathrm{mtRNA}\right)}{P\,\left({\text{data}}_{i}|\mathrm{non}–\mathrm{mtRNA}\right)}\right)\;\text{with}\,\frac{P\,\left({\text{data}}_{i}|\mathrm{mtRNA}\right)}{P\,\left({\text{data}}_{i}|\mathrm{non}–\mathrm{mtRNA}\right)}=\frac{\mathrm{mtRNA}\,\_\,{\mathrm{pos}}_{i}}{\mathrm{mtRNA}\,\_\,{\mathrm{neg}}_{i}}$$

where mtRNA_pos and mtRNA_neg are the fractions of the positive and negative training set genes in sample i, respectively. If there were no training set genes found in a certain bin, that positive or negative training set fraction was set to 0.5/’total number of negative set genes in the complete dataset’ to prevent division by zero and allow calculation of the log ratio. The Oprior, log2(PmtRNA/Pnon-mtRNA), is based on the estimation that 300 of the 20129 protein coding genes encode for a mitochondrial RNA interacting protein, it does not affect the ranking of potential mRNA interacting proteins. Proteins with the same mtRNA score were ranked according to their fold-change in the mtRNA interacting dataset.

To assess the performance a false discovery rate (FDR) was calculated. As this FDR depends on training set genes and these do not resemble the expected number of mtRNA genes versus non-mtRNA interacting genes in the genome, the FDR was corrected (cFDR) using the following formula:

$$\text{cFDR}=\frac{1-\text{specificity}}{1-\mathrm{specificity}+\mathrm{sensitivity}\cdot O_{\text{prior}}}$$

The 211 proteins selected as part of the mtRNA interacting proteome reported in this paper were found at a corrected false discovery rate (cFDR) of 69% (mtRNA score of 1.69). This cut-off score was based on the distribution of training set genes (Figure 5C). The cFDR depends on the chosen cut-off as well as the Oprior (see formula). A higher estimation of the number of mitochondrial RNA interacting proteins would result in a lower cFDR at the same cut-off (an Oprior of 1000 mtRNA interacting proteins would result in a cFDR of 39%). Similarly a cut-off for the top 100 instead of 211 with the same Oprior would result in a cFDR of 31%. Do note that the ranking of the complete human proteome, which does not dependent on the Oprior, is the most informative result of this analysis, proteins high in rank are most likely to interact with mtRNA and can for example be used in conjunction with exome sequencing data from people with congenital mitochondrial disease. However, in order to discuss a specific set of proteins we have to set a cut-off.

### Cross-Validation

To assess the ability of the integrated predictor to discriminate known mtRNA interacting genes from other genes, a ten-fold cross-validation was performed. The training sets (both negative and positive) were subsampled ten times, thereby creating 10 sets of 9/10th of the training sets genes. Each training set gene was left out once in one of the ten sets. Data integration was performed with each of these ten sets and the rank of the one-tenth left out training set genes was retrieved. The ten-fold cross-validated receiver operating characteristic (ROC) curves were constructed based on those ranks.

### Tools for Data Analysis

Plots, statistics and calculations were performed with the R statistical package (R Core Team, 2015) and additional packages gplots (Warnes et al., 2016), ggplot2 (Wickham, 2016), ROCR (Sing et al., 2005), scales (Wickham, 2017), VennDiagram (Chen, 2017) and reshape (Wickham, 2007). Venn diagrams of three circles were made using the web application BioVenn (Hulsen et al., 2008).

## Results

### Validation of Crosslinking Methods

**FIGURE 1:**
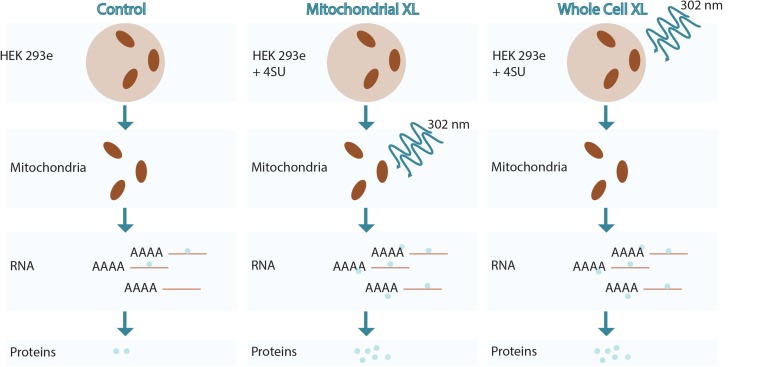
Overview of RNA crosslinking methodologies used.

**FIGURE 2:**
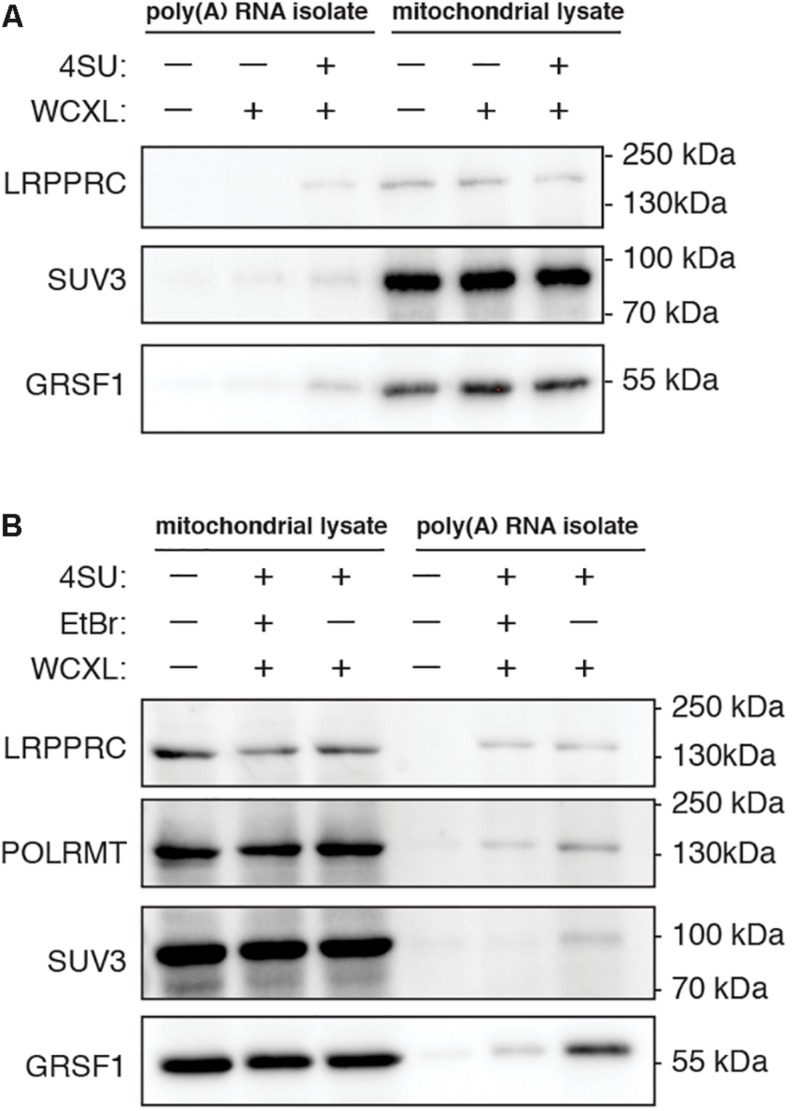
Yield of known mitochondrial RNA interacting proteins after poly(A) RNA isolation is increased when cells are treated with 4-thiouridine prior to whole-cell crosslinking and for some proteins decreased when cells are treated with ethidium bromide. Representative western blots showing the effect on protein yield of **(A)**, 18 h treatment with 100 mM 4-thiouridine (4SU) (*n* = 2), and **(B)**, 4SU treatment either without or with 24 h treatment with 80 ng/mL ethidium bromide (EtBr) (*n* = 2 for LRPPRC, SUV3, and GRSF1, *n* = 1 for POLRMT). To visualize that we start the poly(A) RNA isolation with mitochondrial lysates of equal protein concentration, the mitochondrial lysates prior to poly(A) RNA isolation are shown next to the isolated poly(A) RNA samples. Samples of untreated cells are shown as negative control. Scans of the entire western blot are available in Supplementary Figure S1.

Various methods are conceivable to crosslink proteins to RNA and at the same time enrich for those proteins that are associated with mitochondrial RNA. We focussed on using either WCXL followed by mitochondrial isolation or mitochondrial isolation prior to MXL (Figure 1). We tested and compared these methods for their yield, enrichment and specificity for known mitochondrial RNA interacting proteins. The initial comparison (Figure 2A and Supplementary Figure S1A) included no UV-crosslinking as a negative control and either direct WCXL or WCXL of cells grown in the presence of a photo-activatable ribonucleoside, 4-thio-uridine (4SU) to enhance crosslinking efficiency. We verified these methods by Western blot analysis, testing for several known mitochondrial RNA interacting proteins. These results showed that 4SU labeling resulted in a higher yield of the few examined proteins compared to direct UV-crosslinking.

A second comparison involved normal WCXL with WCXL following treatment with ethidium bromide (WCXL_EtBr), which is expected to rapidly inhibit mitochondrial transcription. Thus, this treatment can be expected to reduce mtRNA binding proteins, while leaving contaminating non-mitochondrial RNA binding proteins unaffected. Using quantitative RT-PCR for several mitochondrial transcripts (Supplementary Figure S2A and Supplementary Table S2), we chose 80 ng/ml ethidium bromide (EtBr) to reduce mitochondrial transcript levels with a 24 h treatment to avoid severe depletion of mtDNA levels. Western blot analysis (Figure 2B and Supplementary Figure S1B) was performed following EtBr treatment and crosslinking with poly(A) RNA isolation in order to compare WCXL_EtBr with WCXL. The results indicate that some mitochondrial RNA interacting proteins, in particular SUV3 and to a lesser extent GRSF1 and POLRMT, are more abundant in the XL sample without EtBr compared to the XL sample with EtBr. But LRPPRC is similar either with or without EtBr treatment.

However, a final statistical comparison of sensitivity and specificity of methods to identify mitochondrial proteins requires a non-targeted approach such as mass spectrometry.

### Mass Spectrometry of Mitochondrial and Whole-Cell Crosslinking Samples

We analyzed isolated poly(A) RNA WCXL and MXL samples using shotgun mass spectrometry analysis of at least three biological replicates and as many technical replicates of each biological replicate and in addition two biological replicates with three technical replicates of isolated poly(A) RNA WCXL_EtBr samples. For each biological replicate, parallel processed non-crosslinked samples were included as controls. After mass spectrometry, label-free quantification values were used to calculate the log2(fold-change) of crosslinked over non-crosslinked samples (MXL and WCXL) or of crosslinked over crosslinked EtBr treated samples (WCXL_EtBr) (see section “Materials and Methods”). Proteins identified with a log2(fold-change) above the log2(3) threshold were considered as enriched.

Mass spectrometry results (Supplementary Table S3) confirmed the Western blot results, and showed that the comparison of EtBr treated cells with non-EtBr treated cells (WCXL_EtBr) identified only 30 enriched annotated mitochondrial RNA-binding proteins compared to 105 with MXL and 62 with WCXL (Supplementary Figure S2B). Thus, for further analysis and the Bayesian integration of various datasets we concentrated on the MXL and WCXL datasets.

**FIGURE 3:**
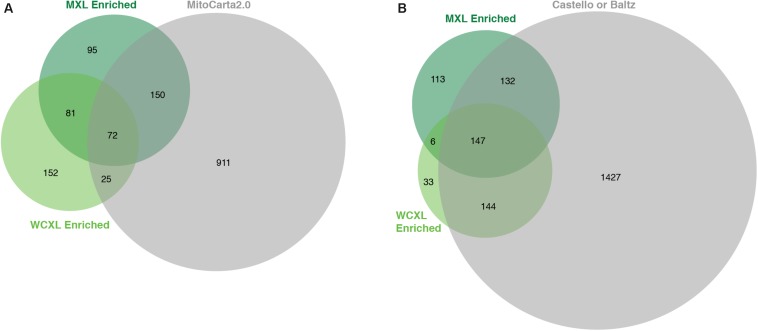
Proteins enriched in our WCXL approach show substantial overlap with published RNA interaction proteomes, but less with a published mitochondrial proteome compared to our MXL approach. **(A)** Proportional Venn diagram showing the overlap of proteins that are identified with a LFQ-intensity fold-change larger than three in the mitochondrial oriented approaches MXL and WCXL with proteins that are part of MitoCarta 2.0 (Calvo et al., 2016). **(B)** Proportional Venn diagram showing the overlap of proteins that are identified with a LFQ-intensity fold-change larger than three in the mitochondrial oriented approaches MXL and WCXL with proteins that are part of either of the complete published whole-cell RNA-interacting proteomes (Baltz et al., 2012; Castello et al., 2012). The fold-changes per proteins were calculated by dividing the average LFQ-values (*n* = 3 for MXL and *n* = 6 for WCXL) of crosslinking with 4SU by non-crosslinking.

**FIGURE 4:**
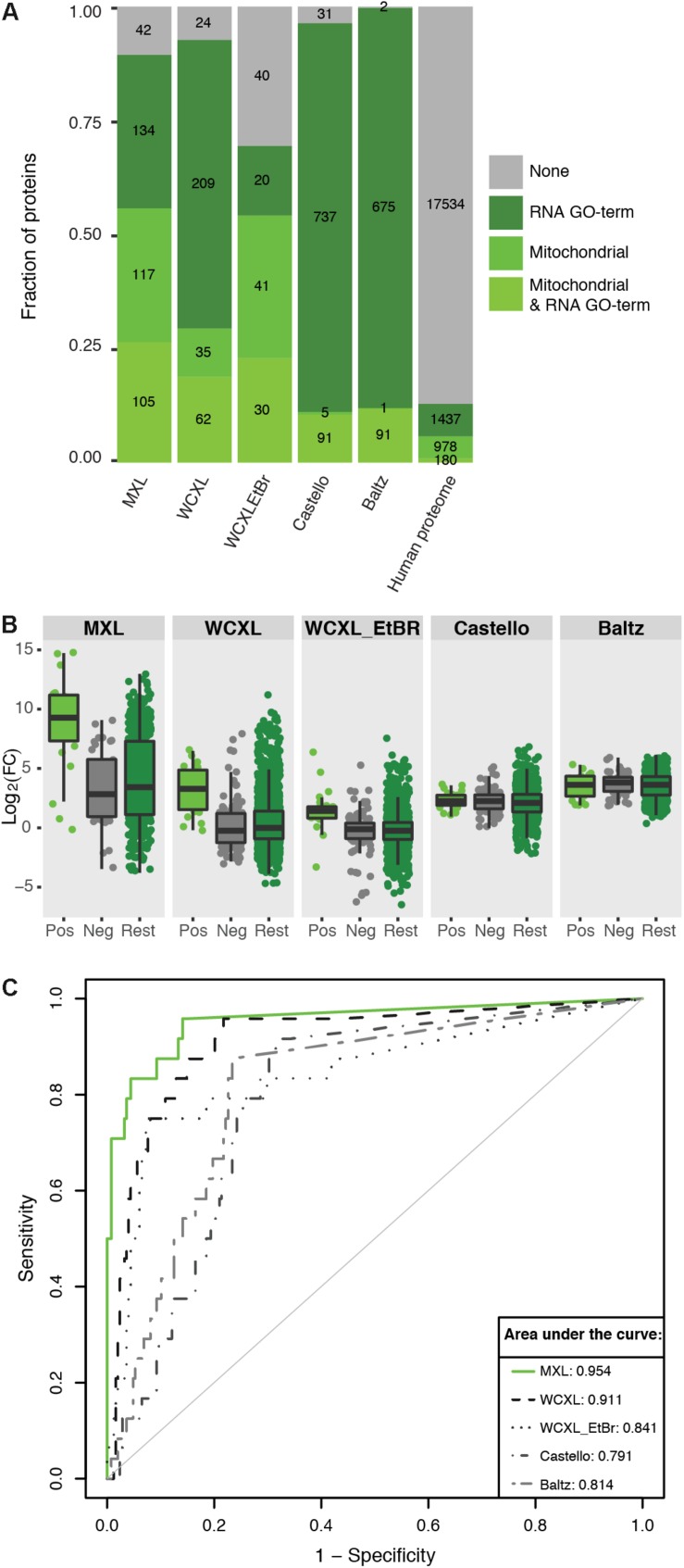
Mitochondria orientated protein-RNA crosslinking approaches enrich for mitochondrial RNA interacting proteins compared to other approaches (Baltz et al., 2012; Castello et al., 2012). **(A)** Barplot showing the fraction and absolute number of proteins identified by our and published approaches (with a LFQ-intensity fold-change larger than three) that are annotated as being mitochondrial (Calvo et al., 2016) and/or interacting with RNA (GO:0003723). The numbers for the complete human proteome are included for reference. **(B)** Boxplot showing the ability of mitochondrial protein-RNA crosslinking approaches to discriminate positive training set members from negative set members based on LFQ-intensity fold-change. “Pos” indicates a manually curated list of 24 mitochondrial RNA binding proteins (no ribosomal proteins included), “Neg” indicates a GO-term based list of 124 mitochondrial non-RNA interacting proteins plus 124 non-mitochondrial RNA interacting proteins, and “Rest” indicate the remaining human proteome (*n* = 19857). **(C)** ROC-curves comparing the performance of the individual approaches to identify mitochondrial RNA interacting proteins. For comparison the AUCs are indicated. The fold-changes per proteins were calculated by dividing the average LFQ-values (*n* = 3 for MXL, *n* = 6 for WCXL and *n* = 2 for WCXL_EtBr) of crosslinking with 4SU by non-crosslinking, or by crosslinking with ethidium bromide and 4SU in case of WCXL_EtBr.

Whole-cell UV-crosslinking identified in total 2.4 times more proteins than MXL (Supplementary Table S3), while the number of enriched proteins with an LFQ difference of at least log2(3) was similar, 330 versus 398, respectively (Figures 3A,B, 4A). From these enriched proteins only 97 are mitochondrial in WXCL, 222 in MXL, with an overlap between the two methods of 72 proteins (Figure 3A). Through comparison with other studies using whole-cell crosslinking (Baltz et al., 2012; Castello et al., 2012), we determined that 291 WCXL enriched proteins and 279 MXL enriched proteins appear to be RNA-binding, with an overlap of 147 proteins (Figure 3B). For MXL, 56% percent of the enriched proteins were mitochondrial and 26% were mitochondrial and had an RNA GO-term, while the fractions for WCXL were 29 and 19%, respectively (Figure 4A).

Volcano plot analysis (Supplementary Figure S3A) resulted in 122 significant proteins in the MXL samples over NoXL samples, with 84 mitochondrial proteins and 89 RNA GO-term containing proteins (overlap was 53 proteins). For WCXL (Supplementary Figure S3B) slightly fewer proteins were significant, 96, of which 92 contained a RNA GO-term and 24 were mitochondrial (overlap was 22 proteins). In total the human proteome contains 180 mitochondrial proteins with an RNA GO-term, so the sensitivity was 0.122 for the WCXL significant list and higher for MXL, namely 0.294. The significant numbers illustrate that information is lost when a statistical test and cut off is applied, e.g., only 83 of the 222 mitochondrial proteins identified with a fold change above three were part of the significant MXL list, and therefore the raw versions of the data [log2(fold-change), also below log2(3)] were used as input for the Bayesian integration (see below), in which the statistics is performed after the data integration.

The above analyses all show that MXL is better in the identification of mitochondrial RNA interacting proteins than WCXL. We nevertheless considered WCXL to be useful since WCXL might better enable the identification of transiently interacting and possibly lowly abundant proteins as crosslinking was applied immediately on the plate prior to cell and mitochondrial isolation. Cell and mitochondrial isolation might cause stress and protein-RNA interactions might be lost or altered because of this, making the detection of lowly abundant proteins even more difficult.

### Defining Conditions for Bayesian Integration

To further quantify the relative value of the different MS datasets for identifying mitochondrial RNA interacting proteins, we used Bayesian data integration. This technique allows combining highly variable datasets by assigning predictive values to each dataset and using those in the final integration. Two training sets were defined in order to calculate the predictive values. (i) As a positive training set, we curated a set of 24 mtRNA interacting proteins from published literature (Supplementary Table S1). (ii) As a negative set, we used GO-terms to select a set of 248 proteins that are highly unlikely to interact with mtRNA, including mitochondrial proteins unlikely to interact with RNA as well as cellular RNA interacting proteins unlikely to be mitochondrial (see section “Materials and Methods). The integration approach then uses the distribution of these training set proteins over the input datasets to define a weighted mtRNA score for all proteins of the human genome indicating the likelihood of its interaction with mtRNA. As final prediction, we integrated independent datasets (Supplementary Figure S4) containing the following features of mtRNA interacting proteins (Table 1); interaction with mtRNA (our MXL and WCXL mass spectrometry data), mitochondrial localization [MitoCarta2.0 (Calvo et al., 2016)], containing RNA binding domain(s) (Gerstberger et al., 2014; Finn et al., 2016), co-expression and protein-protein interaction with mtRNA interacting protein(s) [WEGET (Szklarczyk et al., 2016) and BioPlex 2.0 (Huttlin et al., 2017), respectively]. The bin borders, number of genes in that respective bin and the corresponding mtRNA score are listed in Supplementary Table S4.

### Comparison With Published Whole-Cell RNA Binding Proteomes

In order to compare the enrichment for mitochondrial RNA binding proteins by mitochondrial isolation prior to poly(A) affinity purification with published whole-cell RNA binding proteomes (Baltz et al., 2012; Castello et al., 2012), we had to determine whether a measure of enrichment was shared by all datasets. This was not straightforward as the inclusion criteria for the various categories of the dataset published by Castello et al. (2012) (1758 proteins) were based on either intensity fold-change (FC) of an XL sample over a NoXL sample (available for 1246 proteins) or peptide counts (available for 1739 proteins). The dataset published by Baltz et al. (2012) did not include peptide counts and only includes a fold-change score for proteins with a fold-change larger than three, no data was provided on lower scoring proteins. Thus calculation of a *p*-value adjusted for multiple testing in a similar way for all proteins in each dataset was not possible. Although not all proteins of the Castello dataset have a fold-change score, we decided to use the fold-change of a XL sample over a NoXL sample to compare the methods as this was the only common measure available for all datasets. Thus, using available fold-change values only, we applied several approaches to identify the method that appeared best at predicting mitochondrial poly(A) RNA binding proteins.

The first approach showed distributions of positive and negative training set proteins in the various datasets [i.e., MXL, WCXL, WCXL_EtBr, and, what we hereupon will refer to as the “Castello” dataset (Castello et al., 2012) and the “Baltz” dataset (Baltz et al., 2012)] over log2(FC) values (Figure 4A). This analysis showed that the log2(FC) values of MXL discriminate best between positive and negative training set proteins, followed by the WCXL dataset and the WCXL_EtBr dataset. The other datasets had no discriminative power, which was expected, as these did not apply a mitochondrial enrichment step.

Subsequently, we examined the frequency distribution of four different categories of enriched proteins in each dataset: mitochondrial proteins with an RNA GO-term, remaining mitochondrial proteins, non-mitochondrial proteins with an RNA GO-term and the rest of the proteins (Figure 4B). Enriched proteins are defined as the proteins that are identified with a fold-change of three or larger. This again showed that MXL had the highest enrichment of mitochondrial proteins with, as well as the highest enrichment of mitochondrial proteins without an RNA GO-term. As also shown in Figure 4A this again illustrated that the MXL method yielded the best mitochondrial enrichment also compared to the WCXL method. This was also evident from the volcano plots comparing both these methods (Supplementary Figure S3). Based on these results we expected that both mitochondrial proteins without RNA GO-term and non-mitochondrial proteins with an RNA GO-term in the MXL dataset would have the largest fraction of as yet unidentified mitochondrial proteins with an mtRNA function.

Receiver operating characteristic curves based on the two training sets further confirmed this, again showing that MXL had the best predictive value for mtRNA interacting proteins (Figure 4C). It had an area under the curve (AUC) closest to 1. While MXL performed the best, it in total identified fewer proteins (576) than other datasets (between one and two thousand). WCXL contained information for 1377 proteins and had also a good AUC, therefore MXL and WCXL were combined to form the mtRNA interaction dataset used as input for the Bayesian integration.

### Bayesian Integration Defines a Set of 211 mtRNA Interacting Proteins

**FIGURE 5:**
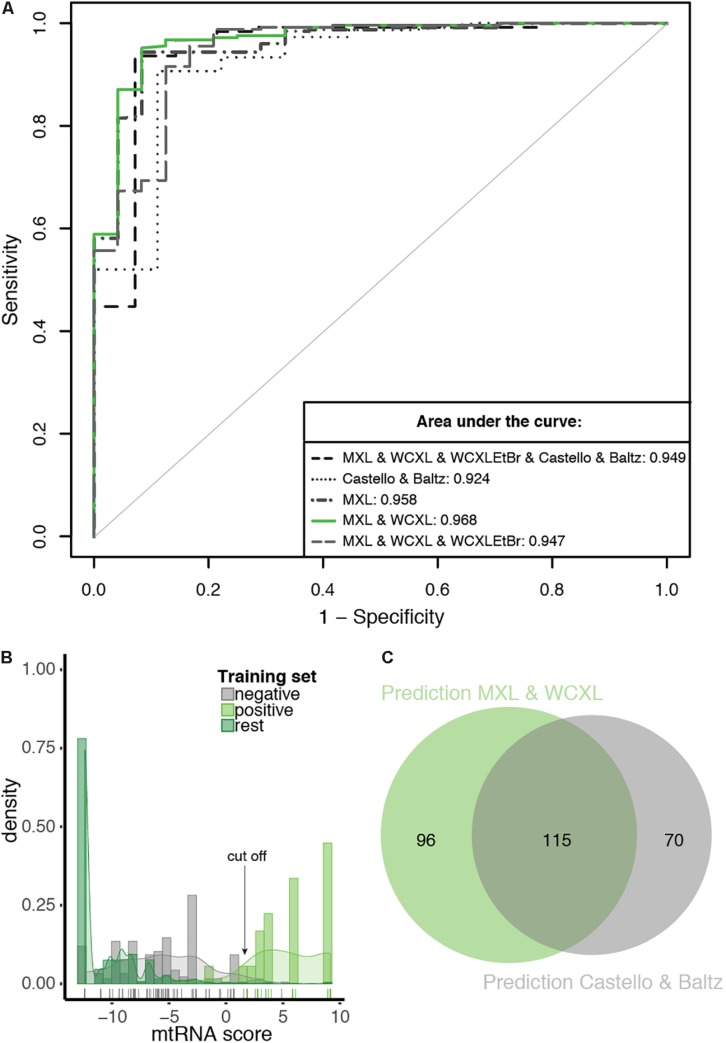
The prediction with the combination of MXL and WCXL as mtRNA interaction input dataset performs best compared to other tested combinations. **(A)** ROC curves of 10-fold cross-validated predictions with as input mitochondrial localization, RNA binding domain, co-expression, PPI and the indicated mtRNA interaction datasets. For comparison the AUCs are indicated. **(B)** Density plot of mtRNA score of the prediction with the combination of MXL and WCXL as mtRNA interaction input, scores of individual training set members are indicated with a colored bar at the bottom of the graph. Arrow indicates the 0.69 cFDR cut-off. **(C)** Proportional Venn diagram showing the overlap of predicted mtRNA interaction proteomes at 0.69 cFDR with the combination of MXL and WCXL as mtRNA interaction input (211 proteins) or with the combination of Castello (Castello et al., 2012) and Baltz (Baltz et al., 2012) (185 proteins).

Based on the analysis above, we pursued Bayesian integration using the combined MXL and WCXL datasets and four other resources as indicated in Table 1. As expected, the integrated score performed best (AUC of 0.968; Supplementary Table S3) when compared with all other RNA-interaction input combinations tested (Figure 5A). The integrated score using the Baltz and Castello datasets gave an AUC 0.924. Even though the Baltz and Castello datasets were suboptimal predictors for mtRNA interacting proteins on their own since they were not specific for mitochondrial proteins, their inclusion in data integration did add predictive power (Figure 5A). With the Bayesian integration using MitoCarta 2.0 as one of the input datasets, in essence we performed an *in silico* enrichment for mitochondrial proteins.

Based on a density plot of the prediction using our MXL and WCXL mass spectrometry data (Figure 5B), we set the cut-off at a cFDR of 69%. This resulted in an mtRNA interacting proteome of 211 proteins at a sensitivity and specificity based on the training sets of 0.958 and 0.968, respectively. Based on the 180 mitochondrial proteins with an RNA GO-term that are part of the human proteome, the sensitivity of the predicted 211 proteins would be 0.539, substantially higher compared to the sensitivity of the significant hits in our mass spectrometry data alone (sensitivity of 0.294 for MXL and 0.122 for WCXL, see above). 185 proteins were identified using Baltz and Castello as input for the prediction and the same cut-off. Our final dataset of 211 proteins and the dataset of 185 proteins based on the published whole-cell RNA binding proteomes showed considerable overlap (Figure 5C), with a total of 115 commonly identified proteins. The frequency distribution of the proteins identified with the integration (Supplementary Figure S5), showed that the proteins identified with the prediction using our mass spectrometry data contained the highest fraction of both mitochondrial and RNA interacting proteins, also when compared with individual datasets (Figure 4B). It is therefore likely that – among the 211 mtRNA proteins – the set of mitochondrial proteins without an RNA GO-term and the set of non-mitochondrial proteins with an RNA GO-term will contain the largest fraction of as yet unidentified mitochondrial proteins with an mtRNA function.

### Identified Proteins

**FIGURE 6:**
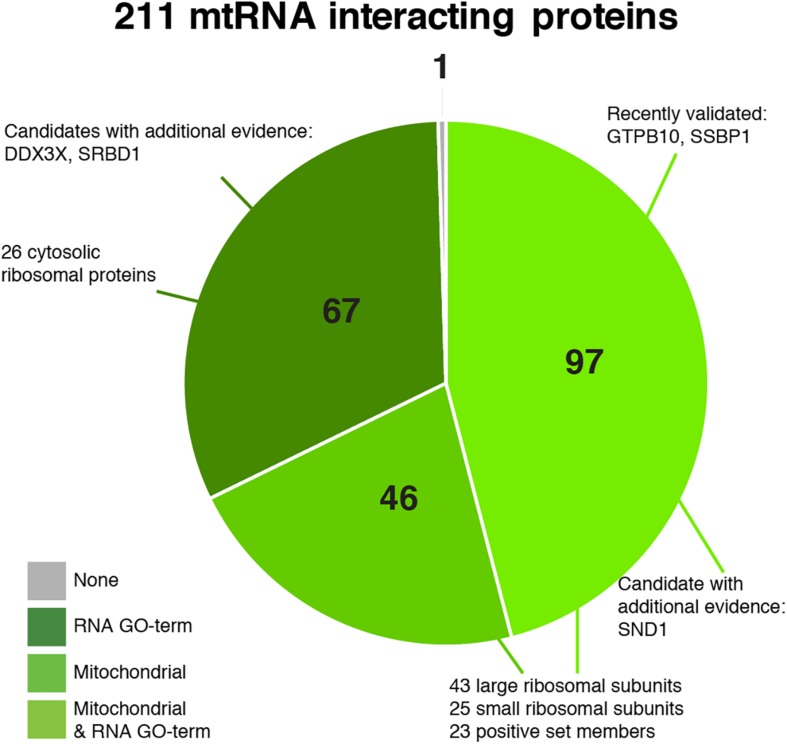
Pie chart of the predicted 211 mitochondrial RNA interacting proteins, divided into categories based on their annotation as being mitochondrial (Calvo et al., 2016) and/or interacting with RNA (GO:0003723).

Our final set of 211 proteins contains 143 proteins that are annotated as mitochondrial in MitoCarta 2.0 (Figure 6, Supplementary Figure S5, and Supplementary Table S3), including 68 mitoribosomal proteins (26 of which have no RNA GO- term), 23 proteins of our positive training set (two of which have no RNA GO-term), and 34 additional proteins that have an RNA GO-term annotation. Forty-six proteins have an established mitochondrial localization and function, but do not have an RNA_GO annotation. An additional 67 proteins are not known as mitochondrial but do have an RNA_GO annotation. Given that our dataset has the highest percentage of mitochondrial proteins (as discussed above), these 67 proteins are candidates with possibly new mitochondrial RNA metabolism related functions.

## Discussion

We developed and compared methods to isolate and identify the mitochondrial poly(A) RNA-binding proteome. Our analyses indicated that 4SU RNA labeling combined with mitochondrial isolation, subsequent crosslinking and poly(A) RNA isolation, scored best in terms of enrichment for mitochondrial RNA binding proteins. It was followed by 4SU labeling, whole-cell crosslinking, and subsequent mitochondrial and poly(A) RNA isolation. Using Bayesian integration we combined our mass spectrometry datasets with publicly available datasets to give likelihood scores to proteins for their mitochondrial RNA association. For the purpose of comparison with published results that used similar methods to identify the whole-cell poly(A) RNA-binding proteome (Baltz et al., 2012; Castello et al., 2012), we used the same Bayesian integration methods and applied thresholds and objective statistical criteria to compare all methods and datasets. These comparisons suggest that by all these criteria, the method of choice to identify mitochondrial RNA binding proteins should be mitochondrial isolation prior to RNA crosslinking and further RNA isolation. Whole-cell crosslinking prior to mitochondrial isolation might inadvertently crosslink RNA containing complexes to the mitochondrial outer membrane, such as cytosolic ribosomes involved in co-translational import of nuclear encoded mitochondrial proteins (Lesnik et al., 2015). It might also alter the ability to isolate mitochondria without too much cytosolic and/or nuclear contamination.

We report a final set of 211 proteins as the predicted mitochondrial RNA interactors, with a sensitivity of 0.958, although the latter might be an overestimate because our training set of 24 known mtRNA interacting proteins was limited. This set includes 67 candidate proteins with an RNA GO-term, but without mitochondrial localization annotation. Nevertheless, these 67 contain potential contaminants, either by their cellular abundance or their association with the mitochondrial outer membrane. Obvious contaminants include a number of cytosolic ribosomal proteins that might be identified on the basis of co-translational import of a substantial fraction of nuclear encoded mitochondrial proteins (Lesnik et al., 2015). Overall, for very few proteins there is additional independent evidence that implies a mitochondrial function. SND1 was annotated to be a possible mitochondrial protein by Zaganelli et al. (2017), while both DDX3X and S1 RNA binding protein 1 (SRBD1) are annotated as possibly mitochondrial in the MitoMiner database (Smith and Robinson, 2016). The mitochondrial localization of SRBD1 was also suggested by immunofluorescence data in Protein Atlas^1^ (Thul et al., 2017). Further testing of these and other candidates could confirm their mitochondrial involvement.

Some proteins identified in our final set have already been validated to be involved in mtRNA metabolism in recent years. The most recent example is GTPBP10 (Lavdovskaia et al., 2018; Maiti et al., 2018), which during the preparation of the current manuscript was reported to be involved in mitoribosomal biogenesis. In addition, one of the surprising proteins identified here is mitochondrial single-stranded DNA-binding protein (mtSSB or SSBP1), which has traditionally been considered as an mtDNA maintenance factor through its role in binding single-stranded DNA during the process of mtDNA replication. Further examination of the function of mtSSB has shown its involvement in mtRNA granules and RNA processing (Hensen et al., 2019). Moreover, in this same study we have shown that it also has bona fide RNA binding activity using EMSA.

Even though we set a threshold to achieve a prioritized list of 211 proteins (cFDR 69%) with possible mitochondrial RNA association, the use of whole cellular genome and proteome datasets in the final Bayesian integration means that we obtained likelihood scores for all proteins in the human proteome (Supplementary Table S3). Because every protein gets a score we can vary the threshold and therewith the cFDR, e.g., a threshold at rank 100 has cFDR of 31% and at rank 150 43%. The value of the cFDR depends in addition on the chosen Oprior of 300 mtRNA interacting proteins, e.g., an Oprior of 1000 mtRNA interacting proteins would result in a cFDR of 39% at rank 211. Note that adjusting the cut-off or the Oprior does not alter the ranking of the proteins, just the estimation of the cFDR. By setting a threshold, information on proteins outside the threshold is discarded, while ultimately the ranking is the most important result from our analysis. Between ranks 211 to 1000 we find additional proteins of interest, including EXD2 (rank 412), evolutionarily conserved signaling intermediate in Toll pathway (ECSIT) (rank 459), C12orf73 (rank 736), and RPUSD2 (rank 982). ECSIT is a known mitochondrial protein associated with respiratory chain complex I (Vogel et al., 2007) and among others is involved in mROS production (West et al., 2011) and mitophagy (Carneiro et al., 2018). Our group found homology of the N-terminus of ECSIT to the (organellar) RNA-binding pentatricopeptide repeat domains (Elurbe and Huynen, 2016), suggesting an additional RNA-binding function. The 3′−5′ exonuclease domain-containing protein 2 (EXD2), acting on both DNA and RNA, was recently published as a mitochondrial protein (Hensen et al., 2018; Silva et al., 2018; Park et al., 2019). Silva et al. reported EXD2 as a mitochondrial matrix or inner membrane ribonuclease involved in among others mitochondrial translation, while Hensen et al. and Park et al. show a mitochondrial outer membrane localization. The other two proteins, uncharacterized protein C12orf73 and RNA pseudouridylate synthase domain-containing protein 2 (RPUSD2) are not known as mitochondrial proteins. Immunofluorescent data of Protein Atlas (see text footnote 1; Thul et al., 2017) suggest among others a mitochondrial localization for C12orf73 and MitoMiner (Smith and Robinson, 2016) predicts a mitochondrial localization for RPUSD2. While there is no molecular function predicted for C12orf73, RPUSD2 is a valid pseudouridine synthase candidate that might be required for RNA modifications (de Crecy-Lagard et al., 2019). The above candidates are but a few examples and illustrate that our ranked list can be considered a valuable resource for anyone that has identified potential mtRNA binding proteins. Zaganelli et al. (2017) used the overlap between the Baltz and Castello datasets with MitoCarta 2.0 to define a set of 207 potential mtRNA interacting proteins. However, in this case no strict statistical or computational framework was used and no selection criteria were applied to the Castello dataset. When we applied the same criteria as used here with our mass spectrometry data in Bayesian integration, we identified 185 proteins using the Baltz and Castello datasets. With the future addition of new datasets as they become available, our predictions should gain further statistical strength, and increase the number of candidate proteins and the likelihood that they interact with mitochondrial mRNAs.

Our current integration relied heavily on proteomics data and, as is the case for many mass spectrometry methods, low abundant and/or transiently interacting proteins could have been missed and therefore score poorly in the final integration. For example, one of our positive set members, MRM1, was not identified in our MXL or WCXL mass spectrometry datasets and therefore did not make the cut-off in our final list of 211 proteins. Furthermore, the approach we chose was directed at poly(A) RNA. This suggests that further improvements in mass spectrometry sensitivity and performing alternative RNA isolation protocols can be expected to further improve our prediction and identify more novel proteins. In addition, further upscaling of the starting material combined with purer mitochondrial fractions can possibly help to identify novel protein candidates. We suggest that MXL should be the method of choice to identify mitochondrial RNA binding proteomes in various cell types and for example tissues from mice for which 4SU exposure prior to mitochondrial isolation is feasible. Alternatively, isolated mitochondria from animal tissues can be directly UV crosslinked without 4SU treatment.

## Data Availability Statement

The mass spectrometry proteomics data have been deposited to the ProteomeXchange Consortium via the PRIDE (Perez-Riverol et al., 2019) partner repository with the dataset identifier PXD014957.

## Author Contributions

MH and JS conceived and designed the study. JS wrote the first draft of the manuscript. SE wrote sections of the manuscript. All authors contributed to the data collection and analysis, manuscript revision, read and approved the submitted version.

## Conflict of Interest

The authors declare that the research was conducted in the absence of any commercial or financial relationships that could be construed as a potential conflict of interest.

## Footnotes

1 www.proteinatlas.org
