# Supplementary material for: A Combined Mass Spectrometry and Data Integration Approach to Predict the Mitochondrial Poly(A) RNA Interacting Proteome
Source: Front Cell Dev Biol. 2019 Nov 15;7:283. doi: 10.3389/fcell.2019.00283 (PMC6873792; doi:10.3389/fcell.2019.00283)
Supplement: Supplementary file 2 [file Data_Sheet_1.pdf]

Supplementary Material

1 Supplementary Materials and Methods

1.1 Quantitative real-time PCR-based analysis (qPCR)

Mitochondrial transcript levels are calculated relative to a nuclear transcript to determine the effect of ethidium bromide treatment on mtRNA. RNA was isolated from ethidium bromide treated and untreated control HEK293e cells by TRIzol (Invitrogen) extraction according to manufacturer's guidelines. A total of 1 µg of RNA was treated with DNase I Amplification Grade (Invitrogen, 18068015) to remove potential mtDNA contamination prior to cDNA synthesis using the SuperScript™ II Reverse Transcriptase (Invitrogen, 18064014) according to manufacturer's protocol with random primers (Promega, C1181), dNTP Mix (Promega, U1515) and with 1 µL Recombinant Rnasin Ribonuclease Inhibitor (Promega, N2515) instead of RNaseOUT™. A minus reverse transcriptase control was taken along for each sample. Absence of mtDNA contamination is tested by a 25 µL regular PCR with DyNAzyme II DNA Polymerase (Thermo Scientific, F501L), dNTP Mix (Promega, U1515) and D-loop or 16S primers (Supplementary Table S2) on 25 ng cDNA or minus reverse transcriptase control followed by 1% agarose gel electrophoresis. Absence of a D-loop PCR band in the cDNA samples and absence of a 16S band in the minus reverse transcriptase samples confirmed that the generated cDNA sample is clean.

Each 20 µL qPCR reaction consists of 25 ng of cDNA, 2.5 mM of forward and reverse primers and 1x SYBR Green Master Mix (Biorad). Measurements were performed in triplicate in Hard-Shell 96-Well PCR Plates (Biorad) within the CFX96 Real-Time System (Biorad). The PCR program consisted of an initial denaturation step at 95°C for 10 minutes followed by 40 cycles of denaturation at 95°C for 15 seconds, primers annealing and extension at 60°C for 60 seconds and fluorescence measurements. The program was followed by a melt-curve analysis to confirm the absence of non-specific amplicons. Primers are listed in Supplementary Table S2. Relative quantities of mitochondrial-encoded transcripts were determined in relation to that of the nuclear-encoded reference gene TATA box binding protein. Changes in the transcripts levels relative to an untreated control were calculated using the $2^{-\Delta\Delta CT}$ method. CT values of technical repeats that varied by more than 0.5 units from the others were removed prior to analysis.

1.2 Mass spectrometry sample preparation

Slightly different tryptic digestion protocols were used for MXL and WCXL approaches. MXL protein samples were subjected to in-solution tryptic digestion as described elsewhere (Wessels et al., 2011). In short the protein sample was diluted 1:1 with 8 M urea, reduced by the addition of 1 µL 10 mM dithiothreitol (30 min at room temperature (RT)) and alkylated by the addition of 1 µL 50 mM chloroacetamide (20 min at RT in dark). Initial digestion with 1 µg LysC (Wako) (3 hours, 37 ^{0}C) was followed by 1:4 dilution with 50 mM ammonium bicarbonate and further digestion with 1 µg sequencing grade modified trypsin (Promega) (overnight, 37 ^{0}C).

WCXL protein samples were subjected to tryptic digestion according to the FASP protocol (Wisniewski et al., 2009) with minor adjustments. In short, all steps (except the DTT step) were performed in one Amicon 3K filters (Merck Millipore, UFC500396) per sample. Concentration or

buffer-exchange steps were performed using centrifugation (16000g for 30 minutes at RT) and flow-through was discarded unless otherwise stated. The WCXL protein sample was reduced in 0.1 M DTT (5 min at 95 ^{o}C), mixed with 200 µL 8 M urea, transferred to and concentrated in a 3K filter. Sample was alkylated with 100 µL 50 mM chloroacetamide (20 min at RT in dark), concentrated and subsequently buffer-exchanged three times with 100 µL 8 M urea. Initial digestion with 1 µg LysC (Wako) in 40 µL 8 M urea (overnight at RT), was followed by digestion with 0.25 µg sequencing grade modified trypsin (Promega) in 120 µL 50 mM ammonium bicarbonate (4 hours at 37 ^{o}C). Filters were transferred to clean collection tubes and the peptides collected by centrifugation (16000g for 30 minutes at RT). An additional 50 µL 0.5 M sodium chloride was added to the filter and left-over peptides collected by a second round of centrifugation (16000g for 30 minutes at RT).

Both MXL and WCXL digested samples were acidified with trifluoroacetic acid to a final concentration of 1%. Thereafter, the peptide sample was desalted and concentrated by "STop And Go Extraction (STAGE) tips" (Rappsilber et al., 2003) and the peptide sample was further purified by Pierce Detergent Removal Spin Columns (Thermo Scientific) before injection in at least triplicate in the mass spectrometer.

1.3 Mass spectrometric measurements

Measurements were performed by nanoLC 1000 (Thermo Scientific) chromatography coupled online to Q Exactive hybrid quadrupole-Orbitrap mass spectrometer (Thermo Scientific). Chromatography was performed with an Acclaim PepMap 0.3 x 5 mm 5µm 100Å trap column (Thermo scientific) in combination with a 15cm long x 100µm ID fused silica electrospray emitter (New Objective, PicoTip Emitter, FS360-100-8-N-5-C15) packed in-house with ReproSil-Pur C18-AQ 3 µm 140Å resin (Dr. Maisch, GMBH) (Ishihama et al., 2002). Tryptic peptides were loaded onto the trap column using 0.1% formic acid and separated by a linear 50 minutes gradient of 5–35% acetonitril containing 0.1% formic acid at a flow rate of 300 nl/min. For MXL a 30 minutes gradient was enough as the MXL samples were less complex. The mass spectrometer was set to positive ion mode. Full MS events were performed at 70.000 resolving power (FWHM) at m/z 200 using 1E6 ions or after 200 ms of maximal injection time (for MXL 20 ms). Data-dependent MS/MS spectra were performed using 1E5 ions at 17.500 resolving power (FWHM) at m/z 200 or after 250 ms maximal injection time (for mMXL 50 ms) for the top 6 precursor ions (for MXL top 10) with an isolation width of 3.0 Th (for MXL 4.0 Th) and fragmented by higher energy collisional dissociation (HCD) with a normalized collision energy of 30%. Dynamic exclusion is set to 10.0 s (for MXL 30.0 s).

1.4 Protein identification

Data analysis was performed with the MaxQuant software (version 1.5.0.25) (Cox and Mann, 2008) applying default settings with minor modifications. The precursor mass tolerance for Q Exactive measurements was set to 4.5 ppm. The multiplicity was set to 1 and Trypsin was chosen as the proteolytic enzyme allowing for 2 miscleavages. Default MaxQuant normalizations were applied. Database searches were performed on the human reference proteome including isoforms in which the reversed database is used to calculate the false discovery rate (FDR) which was set to 1%. Isoleucine and leucine were forced to be treated equally. Between samples the option "Match between runs" was enabled to detect sequenced peptides which were not subjected to sequencing event in other samples and Label-Free Quantification (LFQ) calculation was applied.

1.5 Statistics for Volcano Plots

For statistical analysis the label-free-quantification (LFQ) intensities from the MaxQuant ProteinGroups.txt output file are log2-transformed and included in the analysis when there is a positive LFQ value for at least two of the technical repeats and when the razor and unique peptide count is two or more. Infinite intensities (from missing values) are replaced with the lowest log2(LFQ) measured and the mean of technical repeats is calculated. The difference in log2(LFQ) intensity between crosslinking and non-crosslinking conditions is calculated and statistical tests are performed to produce volcano plots.

2 Supplementary References

Akiyama, T., Gohda, J., Shibata, S., Nomura, Y., Azuma, S., Ohmori, Y., et al. (2001). Mammalian homologue of E. coli Ras-like GTPase (ERA) is a possible apoptosis regulator with RNA binding activity. *Genes Cells* 6(11)**,** 987-1001.

Al-Furoukh, N., Goffart, S., Szibor, M., Wanrooij, S., and Braun, T. (2013). Binding to G-quadruplex RNA activates the mitochondrial GTPase NOA1. *Biochim Biophys Acta* 1833(12)**,** 2933-2942. doi: 10.1016/j.bbamcr.2013.07.022.

Antonicka, H., Sasarman, F., Nishimura, T., Paupe, V., and Shoubridge, E.A. (2013). The mitochondrial RNA-binding protein GRSF1 localizes to RNA granules and is required for posttranscriptional mitochondrial gene expression. *Cell Metab* 17(3)**,** 386-398. doi: 10.1016/j.cmet.2013.02.006.

Antonicka, H., and Shoubridge, E.A. (2015). Mitochondrial RNA Granules Are Centers for Posttranscriptional RNA Processing and Ribosome Biogenesis. *Cell Rep*. doi: 10.1016/j.celrep.2015.01.030.

Beckmann, B.M., Horos, R., Fischer, B., Castello, A., Eichelbaum, K., Alleaume, A.M., et al. (2015). The RNA-binding proteomes from yeast to man harbour conserved enigmRBPs. *Nat Commun* 6**,** 10127. doi: 10.1038/ncomms10127.

Bogenhagen, D.F., Martin, D.W., and Koller, A. (2014). Initial steps in RNA processing and ribosome assembly occur at mitochondrial DNA nucleoids. *Cell Metab* 19(4)**,** 618-629. doi: 10.1016/j.cmet.2014.03.013.

Brown, T.A., Tkachuk, A.N., and Clayton, D.A. (2015). Mitochondrial Transcription Factor A (TFAM) Binds to RNA Containing 4-Way Junctions and Mitochondrial tRNA. *PLoS One* 10(11)**,** e0142436. doi: 10.1371/journal.pone.0142436.

Brzezniak, L.K., Bijata, M., Szczesny, R.J., and Stepien, P.P. (2011). Involvement of human ELAC2 gene product in 3' end processing of mitochondrial tRNAs. *RNA Biol* 8(4)**,** 616-626. doi: 10.4161/rna.8.4.15393.

Calvo, S.E., Clauser, K.R., and Mootha, V.K. (2016). MitoCarta2.0: an updated inventory of mammalian mitochondrial proteins. *Nucleic Acids Res* 44(D1)**,** D1251-1257. doi: 10.1093/nar/gkv1003.

Chen, H.W., Rainey, R.N., Balatoni, C.E., Dawson, D.W., Troke, J.J., Wasiak, S., et al. (2006). Mammalian polynucleotide phosphorylase is an intermembrane space RNase that maintains mitochondrial homeostasis. *Mol Cell Biol* 26(22)**,** 8475-8487. doi: 10.1128/mcb.01002-06.

Chujo, T., Ohira, T., Sakaguchi, Y., Goshima, N., Nomura, N., Nagao, A., et al. (2012). LRPPRC/SLIRP suppresses PNPase-mediated mRNA decay and promotes polyadenylation in human mitochondria. *Nucleic Acids Res* 40(16)**,** 8033-8047. doi: 10.1093/nar/gks506.

Cox, J., and Mann, M. (2008). MaxQuant enables high peptide identification rates, individualized p.p.b.-range mass accuracies and proteome-wide protein quantification. *Nat Biotechnol* 26(12)**,** 1367-1372. doi: 10.1038/nbt.1511.

Das, S.K., Sokhi, U.K., Bhutia, S.K., Azab, B., Su, Z.Z., Sarkar, D., et al. (2010). Human polynucleotide phosphorylase selectively and preferentially degrades microRNA-221 in human melanoma cells. *Proc Natl Acad Sci U S A* 107(26)**,** 11948-11953. doi: 10.1073/pnas.0914143107.

Davies, S.M., Rackham, O., Shearwood, A.M., Hamilton, K.L., Narsai, R., Whelan, J., et al. (2009). Pentatricopeptide repeat domain protein 3 associates with the mitochondrial small ribosomal subunit and regulates translation. *FEBS Lett* 583(12)**,** 1853-1858. doi: 10.1016/j.febslet.2009.04.048.

Dennerlein, S., Rozanska, A., Wydro, M., Chrzanowska-Lightowlers, Z.M., and Lightowlers, R.N. (2010). Human ERAL1 is a mitochondrial RNA chaperone involved in the assembly of the 28S small mitochondrial ribosomal subunit. *Biochem J* 430(3)**,** 551-558. doi: 10.1042/bj20100757.

Hatchell, E.C., Colley, S.M., Beveridge, D.J., Epis, M.R., Stuart, L.M., Giles, K.M., et al. (2006). SLIRP, a small SRA binding protein, is a nuclear receptor corepressor. *Mol Cell* 22(5)**,** 657-668. doi: 10.1016/j.molcel.2006.05.024.

He, J., Cooper, H.M., Reyes, A., Di Re, M., Kazak, L., Wood, S.R., et al. (2012). Human C4orf14 interacts with the mitochondrial nucleoid and is involved in the biogenesis of the small mitochondrial ribosomal subunit. *Nucleic Acids Res* 40(13)**,** 6097-6108. doi: 10.1093/nar/gks257.

Ishihama, Y., Rappsilber, J., Andersen, J.S., and Mann, M. (2002). Microcolumns with self-assembled particle frits for proteomics. *J Chromatogr A* 979(1-2)**,** 233-239.

Jedrzejczak, R., Wang, J., Dauter, M., Szczesny, R.J., Stepien, P.P., and Dauter, Z. (2011). Human Suv3 protein reveals unique features among SF2 helicases. *Acta Crystallogr D Biol Crystallogr* 67(Pt 11)**,** 988-996. doi: 10.1107/s0907444911040248.

Kuhl, I., Kukat, C., Ruzzenente, B., Milenkovic, D., Mourier, A., Miranda, M., et al. (2014). POLRMT does not transcribe nuclear genes. *Nature* 514(7521)**,** E7-11. doi: 10.1038/nature13690.

Lagouge, M., Mourier, A., Lee, H.J., Spahr, H., Wai, T., Kukat, C., et al. (2015). SLIRP Regulates the Rate of Mitochondrial Protein Synthesis and Protects LRPPRC from Degradation. *PLoS Genet* 11(8)**,** e1005423. doi: 10.1371/journal.pgen.1005423.

Lee, K.W., and Bogenhagen, D.F. (2014). Assignment of 2'-O-methyltransferases to modification sites on the mammalian mitochondrial large subunit 16 S ribosomal RNA (rRNA). *J Biol Chem* 289(36)**,** 24936-24942. doi: 10.1074/jbc.C114.581868.

Lee, K.W., Okot-Kotber, C., LaComb, J.F., and Bogenhagen, D.F. (2013). Mitochondrial ribosomal RNA (rRNA) methyltransferase family members are positioned to modify nascent rRNA in

foci near the mitochondrial DNA nucleoid. *J Biol Chem* 288(43)**,** 31386-31399. doi: 10.1074/jbc.M113.515692.

Minczuk, M., Mroczek, S., Pawlak, S.D., and Stepien, P.P. (2005). Human ATP-dependent RNA/DNA helicase hSuv3p interacts with the cofactor of survivin HBXIP. *Febs j* 272(19)**,** 5008-5019. doi: 10.1111/j.1742-4658.2005.04910.x.

Nagaike, T., Suzuki, T., Katoh, T., and Ueda, T. (2005). Human mitochondrial mRNAs are stabilized with polyadenylation regulated by mitochondria-specific poly(A) polymerase and polynucleotide phosphorylase. *J Biol Chem* 280(20)**,** 19721-19727. doi: 10.1074/jbc.M500804200.

Piwowarski, J., Grzechnik, P., Dziembowski, A., Dmochowska, A., Minczuk, M., and Stepien, P.P. (2003). Human polynucleotide phosphorylase, hPNPase, is localized in mitochondria. *J Mol Biol* 329(5)**,** 853-857.

Portnoy, V., Palnizky, G., Yehudai-Resheff, S., Glaser, F., and Schuster, G. (2008). Analysis of the human polynucleotide phosphorylase (PNPase) reveals differences in RNA binding and response to phosphate compared to its bacterial and chloroplast counterparts. *Rna* 14(2)**,** 297-309. doi: 10.1261/rna.698108.

Rackham, O., Davies, S.M., Shearwood, A.M., Hamilton, K.L., Whelan, J., and Filipovska, A. (2009). Pentatricopeptide repeat domain protein 1 lowers the levels of mitochondrial leucine tRNAs in cells. *Nucleic Acids Res* 37(17)**,** 5859-5867. doi: 10.1093/nar/gkp627.

Rappsilber, J., Ishihama, Y., and Mann, M. (2003). Stop and go extraction tips for matrix-assisted laser desorption/ionization, nanoelectrospray, and LC/MS sample pretreatment in proteomics. *Anal Chem* 75(3)**,** 663-670.

Rossmanith, W. (2011). Localization of human RNase Z isoforms: dual nuclear/mitochondrial targeting of the ELAC2 gene product by alternative translation initiation. *PLoS One* 6(4)**,** e19152. doi: 10.1371/journal.pone.0019152.

Sanchez, M.I., Mercer, T.R., Davies, S.M., Shearwood, A.M., Nygard, K.K., Richman, T.R., et al. (2011). RNA processing in human mitochondria. *Cell Cycle* 10(17)**,** 2904-2916. doi: 10.4161/cc.10.17.17060.

Sasarman, F., Brunel-Guitton, C., Antonicka, H., Wai, T., and Shoubridge, E.A. (2010). LRPPRC and SLIRP interact in a ribonucleoprotein complex that regulates posttranscriptional gene expression in mitochondria. *Mol Biol Cell* 21(8)**,** 1315-1323. doi: 10.1091/mbc.E10-01-0047.

Schwinghammer, K., Cheung, A.C., Morozov, Y.I., Agaronyan, K., Temiakov, D., and Cramer, P. (2013). Structure of human mitochondrial RNA polymerase elongation complex. *Nat Struct Mol Biol* 20(11)**,** 1298-1303. doi: 10.1038/nsmb.2683.

Szczesny, R.J., Borowski, L.S., Brzezniak, L.K., Dmochowska, A., Gewartowski, K., Bartnik, E., et al. (2010). Human mitochondrial RNA turnover caught in flagranti: involvement of hSuv3p helicase in RNA surveillance. *Nucleic Acids Res* 38(1)**,** 279-298. doi: 10.1093/nar/gkp903.

Tomecki, R., Dmochowska, A., Gewartowski, K., Dziembowski, A., and Stepien, P.P. (2004). Identification of a novel human nuclear-encoded mitochondrial poly(A) polymerase. *Nucleic Acids Res* 32(20)**,** 6001-6014. doi: 10.1093/nar/gkh923.

Vilardo, E., Nachbagauer, C., Buzet, A., Taschner, A., Holzmann, J., and Rossmanith, W. (2012). A subcomplex of human mitochondrial RNase P is a bifunctional methyltransferase--extensive

moonlighting in mitochondrial tRNA biogenesis. *Nucleic Acids Res* 40(22)**,** 11583-11593. doi: 10.1093/nar/gks910.

Wang, G., Chen, H.W., Oktay, Y., Zhang, J., Allen, E.L., Smith, G.M., et al. (2010). PNPASE regulates RNA import into mitochondria. *Cell* 142(3)**,** 456-467. doi: 10.1016/j.cell.2010.06.035.

Wessels, H.J., Gloerich, J., van der Biezen, E., Jetten, M.S., and Kartal, B. (2011). Liquid chromatography-mass spectrometry-based proteomics of Nitrosomonas. *Methods Enzymol* 486**,** 465-482. doi: 10.1016/b978-0-12-381294-0.00021-3.

Wisniewski, J.R., Zougman, A., Nagaraj, N., and Mann, M. (2009). Universal sample preparation method for proteome analysis. *Nat Methods* 6(5)**,** 359-362. doi: 10.1038/nmeth.1322.

Wolf, A.R., and Mootha, V.K. (2014). Functional genomic analysis of human mitochondrial RNA processing. *Cell Rep* 7(3)**,** 918-931. doi: 10.1016/j.celrep.2014.03.035.

3 Supplementary Figure Captions and Tables

Supplementary Figure 1. Yield of known mitochondrial RNA interacting proteins after poly(A) RNA isolation is increased when cells are treated with 4-thiouridine prior to whole-cell crosslinking and for some proteins decreased when cells are treated with ethidium bromide. Scans of uncropped representative western blots (used to create Figure 2) showing the effect on protein yield of **(A)**, 18 hour treatment with 100 mM 4-thiouridine (4SU) (n=2), and **(B)**, 4SU treatment either without or with 24 hour treatment with 80 ng/mL ethidium bromide (EtBr) (n=2 for LRPPRC, SUV3 and GRSF1, n=1 for POLRMT). To visualize that we start the poly(A) RNA isolation with mitochondrial lysates of equal protein concentration, the mitochondrial lysates prior to poly(A) RNA isolation are shown next to the isolated poly(A) RNA samples. Samples of untreated cells are shown as negative control. For this figure the scans were merged with an epiwhite picture of the blot to visualize the edges of the blot and the used marker, for Figure 2 the non-merged scans were used. The order of the pictures matches with the order of probing the blot with the specific antibodies.

Supplementary Figure 2. The effect of ethidium bromide treatment. (A) Quantitative RT-PCR analysis of mitochondrial transcripts levels after 18 hour ethidium bromide treatment relative to untreated cells (n=1). (B) Proportional Venn diagram showing the overlap of proteins that are identified with a LFQ-intensity fold-change larger than 3 in the three mitochondrial oriented approaches MXL, WCXL and WCXL_EtBr. Only proteins that have a RNA GO-term (GO:0003723) and are part of MitoCarta2.0 (Calvo et al., 2016) are included in the diagram. The fold-changes per proteins were calculated by dividing the average LFQ-values (n=3 for MXL, n=6 for WCXL and n=2 for WCXL_EtBr) of crosslinking with 4SU by non-crosslinking, or by crosslinking with ethidium bromide and 4SU in case of WCXL_EtBr.

Supplementary Figure 3. MXL approach enriches better for mitochondrial as well as mitochondrial RNA interacting proteins compared to WCXL approach. Volcano plots showing significant detected proteins (two-sided t-test with μ=0, corrected for multiple testing with Benjamini Hochberg, FDR 10%) in the MXL approach (A, n=3) and WCXL approach (B, n=6) compared to non-crosslinking samples (NoXL). All proteins above the red dotted lines are significant. Light green round dot represent proteins that are associated with the molecular function RNA GO-term (GO:0003723) and are part of the MitoCarta2.0 compendium (Calvo et al., 2016), dark green triangles represent

remaining proteins that are part of MitoCarta2.0, black squares represent remaining proteins that have the RNA GO term and grey plus signs represent remaining proteins.

Supplementary Figure 4. Correlations between the five input datasets used for the final Bayesian prediction. Heatmaps depict pairwise Spearman’s rank correlation coefficients.

Supplementary Figure 5. Prediction with MXL and WCXL as input dataset enriches best for proteins that are mitochondrial. Barplot plot showing the fraction and absolute number of proteins predicted to interact with mtRNA that are annotated as being mitochondrial (Calvo et al., 2016) and/or interacting with RNA (GO:0003723). Results for the predictions with either the combination of MXL and WCXL or Castello and Baltz as mtRNA interaction input dataset are shown. As a reference the numbers for the complete human proteome are included.

Supplementary Table S1. Positive training set proteins are listed with the reference(s) showing interaction with mtRNA.

Gene	Uniprot	Description	Reference
KIAA0391	O15091	Mitochondrial ribonuclease P catalytic subunit	(Sanchez et al., 2011)
ELAC2	Q9BQ52	elaC ribonuclease Z 2	(Brzezniak et al., 2011; Rossmanith, 2011; Sanchez et al., 2011)
MRM2	Q9UI43	mitochondrial rRNA methyltransferase 2	(Lee et al., 2013; Lee and Bogenhagen, 2014)
MRM3	Q9HC36	mitochondrial rRNA methyltransferase 3	(Lee et al., 2013; Lee and Bogenhagen, 2014)
MRM1	Q6IN84	mitochondrial rRNA methyltransferase 1	(Lee et al., 2013; Lee and Bogenhagen, 2014)
MTPAP	Q9NVV4	mitochondrial poly(A) polymerase	(Tomecki et al., 2004; Nagaike et al., 2005)
TBRG4	Q969Z0	transforming growth factor beta regulator 4	(Wolf and Mootha, 2014)
LRPPRC	P42704	leucine rich pentatricopeptide repeat containing	(Sasarman et al., 2010; Chujo et al., 2012; Wolf and Mootha, 2014; Lagouge et al., 2015)
GRSF1	Q12849	G-rich RNA sequence binding factor 1	(Antonicka et al., 2013; Antonicka and Shoubridge, 2015)
DHX30	Q7L2E3	DExH-box helicase 30	(Antonicka and Shoubridge, 2015)
DDX28	Q9NUL7	DEAD-box helicase 28	(Antonicka and Shoubridge, 2015)
FASTKD2	Q9NYY8	FAST kinase domains 2	(Antonicka and Shoubridge, 2015)
FASTKD5	Q7L8L6	FAST kinase domains 5	(Antonicka and Shoubridge, 2015)
ERAL1	O75616	Era like 12S mitochondrial rRNA chaperone 1	(Akiyama et al., 2001; Dennerlein et al., 2010)
POLRMT	O00411	RNA polymerase mitochondrial	(Schwinghammer et al., 2013; Kuhl et al., 2014)
PNPT1	Q8TCS8	polyribonucleotide nucleotidyltransferase 1	(Piwowarski et al., 2003; Chen et al., 2006; Portnoy et al., 2008; Das et al., 2010; Wang et al., 2010)
SUPV3L1	Q8IYB8	Suv3 like RNA helicase	(Minczuk et al., 2005; Szczesny et al., 2010; Jedrzejczak et al., 2011)
NOA1	Q8NC60	nitric oxide associated 1	(He et al., 2012; Al-Furoukh et al., 2013)
TFAM	Q00059	transcription factor A, mitochondrial	(Brown et al., 2015)
HSD17B10	Q99714	hydroxysteroid 17-beta dehydrogenase 10, mitochondrial RNase P subunit	(Bogenhagen et al., 2014; Beckmann et al., 2015)
TRMT10C	Q7L0Y3	tRNA methyltransferase 10C, mitochondrial RNase P subunit	(Sanchez et al., 2011; Vilardo et al., 2012)
SLIRP	Q9GZT3	SRA stem-loop interacting RNA binding protein	(Hatchell et al., 2006)
PTCD1	O75127	pentatricopeptide repeat domain 1	(Rackham et al., 2009; Sanchez et al., 2011)
PTCD3	Q96EY7	pentatricopeptide repeat domain 3	(Davies et al., 2009)

Supplementary Table S2. Primers used in this manuscript.

Primer	Sequence (5' → 3')
35L (D-loop, F)	GGA GCT CTC CAT GCA TTT GG
611H (D-loop,R)	CAG TGT ATT GCT TTG AGG AGG
TATA box binding protein, F	GCT GGC CCA TAG TGA TCT TT
TATA box binding protein, R	TCC TTG GGT TAT CTT CAC ACG
h16S-F	GGT AGA GGC GAC AAA CCT ACC G
h16S-R	CAG GCG GGG TAA GAT TTG CCG AG
hND2-F	GGC CCA ACC CGT CAT CTA C
hND2-R	GGG TTT GGT TTA ATC CAC
hCOXI-F	GGC GCA TGA GCT GGA GTC
hCOXI-R	GCT GTG ATT AGG ACG GAT C
hND5-F	CAG TCT CAG CCC TAC TCC AC
hND5-R	GAA GCG AGG TTG ACC TGT TAG

Supplementary Table S4. Bin borders, amount of genes in that respective bin and the corresponding mtRNA score

Dataset	Bin_borders	mtRNA score (log-likelihood)	Total number of genes
RNA interaction	[NA,-5.19]	-3.728798273	18705
	(-5.19,3.5]	-1.27462238	1149
	(3.5,11.1]	4.157729704	275
Mitochondrial localization	[-13,-3]	-3.438121112	16045
	(-3,5]	0.614346308	1942
	(5,44]	0.848976998	978
RNA binding domain	[0,0.99]	-1.718229032	18589
	(0.99,1]	1.331759104	1520
Co-expression with mtRNA interactors	[0,1.5e+03]	0.816964916	1500
	(1.5e+03,3e+03]	-0.801356181	1481
	(3e+03,2.2e+04]	-2.195635121	15230
PPI with mtRNA interactors	[0,0.99]	-1.324253148	18729
	(0.99,35]	2.011681805	1400
