# Supplementary figures and images for: A Combined Mass Spectrometry and Data Integration Approach to Predict the Mitochondrial Poly(A) RNA Interacting Proteome
Source: Front Cell Dev Biol. 2019 Nov 15;7:283. doi: 10.3389/fcell.2019.00283 (PMC6873792; doi:10.3389/fcell.2019.00283)

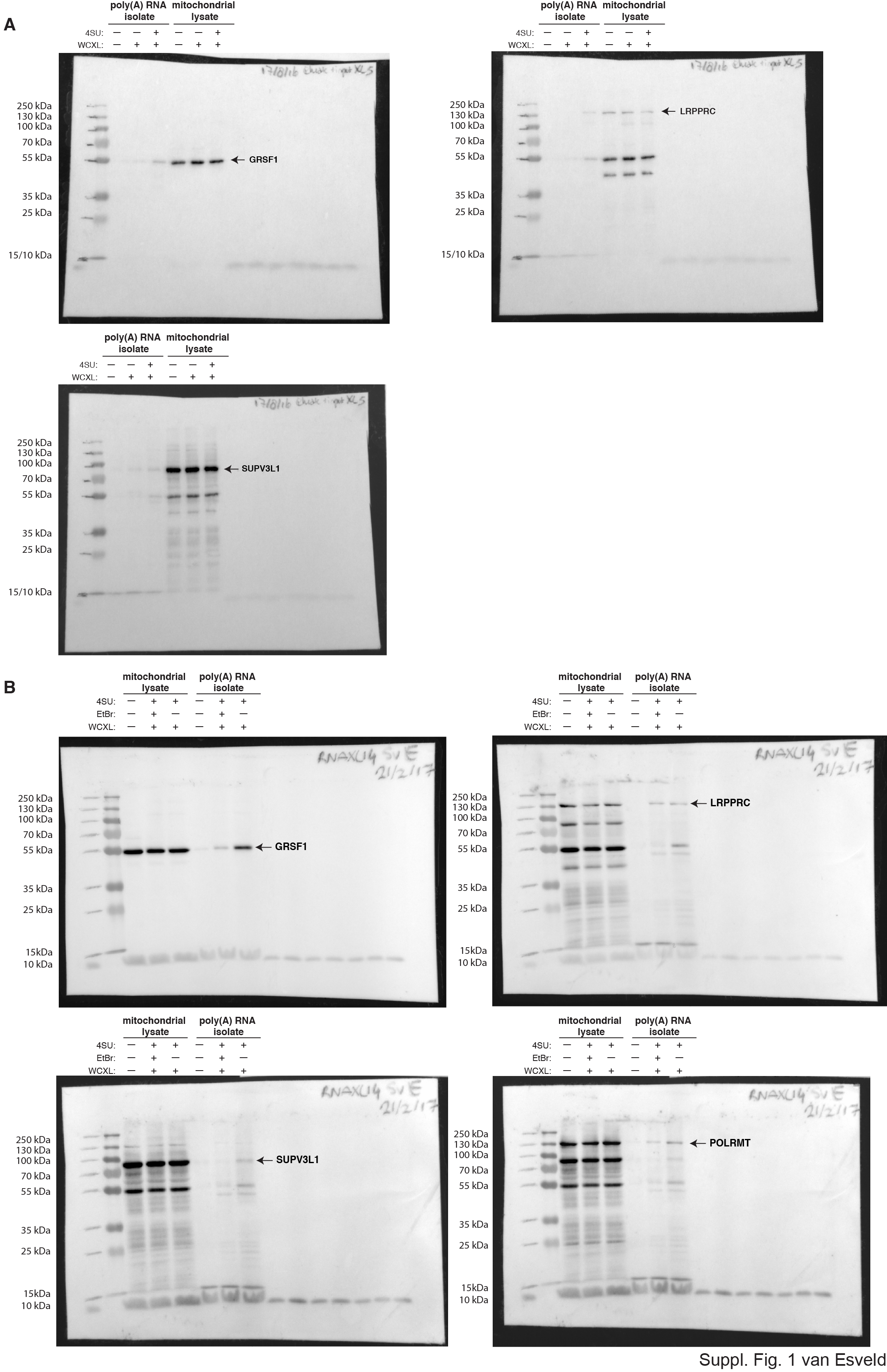

Supplement: Supplementary file 3 [file Image_1.TIF]
